# Comprehensive Analysis and Identification of Key Driver Genes for Distinguishing Between Esophageal Adenocarcinoma and Squamous Cell Carcinoma

**DOI:** 10.3389/fcell.2021.676156

**Published:** 2021-05-28

**Authors:** Feng Wang, Lan Zhang, Yue Xu, Yilin Xie, Shenglei Li

**Affiliations:** ^1^Department of Oncology, The First Affiliated Hospital of Zhengzhou University, Zhengzhou, China; ^2^Department of Pathology, The First Affiliated Hospital, Zhengzhou University, Zhengzhou, China

**Keywords:** esophageal adenocarcinoma, esophageal squamous cell carcinoma, hub genes, prognosis, biomarkers

## Abstract

**Background:** Esophageal cancer (EC) is one of the deadliest cancers in the world. However, the mechanism that drives the evolution of EC is still unclear. On this basis, we identified the key genes and molecular pathways that may be related to the progression of esophageal adenocarcinoma and squamous cell carcinoma to find potential markers or therapeutic targets.

**Methods:** GSE26886 were obtained from Gene Expression Omnibus (GEO) database. The differentially expressed genes (DEGs) among normal samples, EA, and squamous cell carcinoma were determined using R software. Then, potential functions of DEGs were determined using the Database for Annotation, Visualization and Integrated Discovery (DAVID). The STRING software was used to identify the most important modules in the protein–protein interaction (PPI) network. The expression levels of hub genes were confirmed using UALCAN database. Kaplan–Meier plotters were used to confirm the correlation between hub genes and outcomes in EC.

**Results:** In this study, we identified 1,098 genes induced in esophageal adenocarcinoma (EA) and esophageal squamous cell carcinoma (ESCC), and 669 genes were reduced in EA and ESCC, suggesting that these genes may play an important role in the occurrence and development of EC tumors. Bioinformatics analysis showed that these genes were involved in cell cycle regulation and p53 and phosphoinositide 3-kinase (PI3K)/Akt signaling pathway. In addition, we identified 147 induced genes and 130 reduced genes differentially expressed in EA and ESCC. The expression of ESCC in the EA group was different from that in the control group. By PPI network analysis, we identified 10 hub genes, including GNAQ, RGS5, MAPK1, ATP1B1, HADHA, HSDL2, SLC25A20, ACOX1, SCP2, and NLN. TCGA validation showed that these genes were present in the dysfunctional samples between EC and normal samples and between EA and ESCC. Kaplan–Meier analysis showed that MAPK1, ACOX1, SCP2, and NLN were associated with overall survival in patients with ESCC and EA.

**Conclusions:** In this study, we identified a series of DEGs between EC and normal samples and between EA and ESCC samples. We also identified 10 key genes involved in the EC process. We believe that this study may provide a new biomarker for the prognosis of EA and ESCC.

## Introduction

According to the cancer statistics in 2018, the mortality rate of esophageal cancer ranks sixth among all tumors all over the world (Bray et al., 2018; Gu et al., 2020b). Esophageal carcinoma (EC) is divided into esophageal adenocarcinoma (EA) and esophageal squamous cell carcinoma (ESCC) (Then et al., 2020). ESCC mostly occurs in the upper and middle portions of the esophagus and related to alcohol and nicotine abuse (Then et al., 2020). ESCC is particularly prominent in China, accounting for about 88% of EC (Wang et al., 2014). Esophageal adenocarcinoma is a highly invasive histological subtype, which is dominant in western countries (Abbas and Krasna, 2017). EA occurs in the lower portion of the esophagus and arises as a consequence of persistent gastroesophageal reflux from areas with specialized intestinal metaplasia in Barrett’s esophagus (Gindea et al., 2014), The 5-year survival rate is as low as 20% (Abbas and Krasna, 2017). At present, the treatment methods of the two EC are similar, including chemotherapy, radiotherapy, and surgery, in which surgery is the most common treatment (Kelsen et al., 1998). Identifying biomarkers for EC development, progression, and prognosis is essential for understanding EC and improving clinical decision-making.

In the past few decades, a large number of studies have revealed the potential mechanism of regulating EC progression. For example, N-myc-downregulated gene 4 (NDRG4) plays a role in cancer suppression of EA (Cao et al., 2020). Inhibition of DCLK1 can reduce the incidence of EC and improve its chemosensitivity by inhibiting β-catenin/c-myc signal (Whorton et al., 2015; Zhang et al., 2020). Notch signal pathway mediates Barrett’s esophageal differentiation and promotes its development to adenocarcinoma (Kunze et al., 2020). Abnormal WNT5A/ROR2 signaling pathway is a characteristic of Barrett-related EA (Lyros et al., 2016). At the same time, multiple bioinformatics analysis of EC was carried out based on RNA sequences and microarray datasets (Zhang H. et al., 2019). For example, a total of 345 DEGs were identified by Zhang H. et al. (2019) in normal esophageal and ESCC samples, including Kyoto Encyclopedia of Genes and Genomes (KEGG) pathway of endocytosis, pancreatic secretion, and fatty acids. However, the regulatory mechanism in EC is still not clear.

In this study, we downloaded GSE26886 (Wang et al., 2013) from the Gene Expression Omnibus (GEO) database. DEGs among esophageal squamous epithelium, Barrett’s esophagus, EA, and ESCC were analyzed. Then, the KEGG pathway and protein–protein interaction (PPI) network of DEG are analyzed. Finally, the survival rate of the identified core gene was verified and analyzed. The core gene may be a novel biomarker and therapeutic target for esophageal cancer.

## Materials and Methods

### GEO Gene Expression Data

In this study, we aimed to identify differently expressed specific biomarkers to distinguished EA from ESCC. By screening GEO datasets, only GSE26886 include four types of EC-related samples, including healthy controls, Barrett’s esophagus, EA, and ESCC, thus selected for further analysis. GSE26886 (Wang et al., 2013) were obtained from the GEO database. A total of 69 frozen specimens were collected, including 19 healthy controls, 20 Barrett’s esophagus, 21 EA, and 9 ESCC.

### Data Processing and DEGs Filtering

The Database for Annotation, Visualization, and Integrated Discovery (DAVID) 6.8 (Huang da et al., 2009) was used to analyze the GO function of integrating DEG and KEGG paths (Shi et al., 2018b; Gu et al., 2020d, 2021). GO term and KEGG pathways with *P* < 0.05 were selected as enrichment functions (Gu et al., 2020c).

### PPI Network Analysis

Protein–protein interaction network is an online tool for building data from STRINGS^1^. The platform reveals protein interaction and functional analysis (Shi et al., 2018a; Shi X. et al., 2020). The most important modules in the PPI network were identified by insertion molecular complex detection (MCODE) with criteria (Shannon et al., 2003): degree value = 2, node score value = 0.2, and K score = 2. Then, the GO function and KEGG pathway of genes in these modules were using DAVID, with statistical significance (*P* < 0.05).

### Validation of Hub Genes in EC

UALCAN^2^ data were analyzed to compare the expression of hub gene in esophageal squamous epithelium, Barrett’s esophagus, EA, and ESCC (Chandrashekar et al., 2017). Gene expression profile interaction analysis (GEPIA) (Tang et al., 2017; Gu et al., 2020a) was used to analyze the overall survival curve of each key gene, where *P* < 0.05 was considered to be statistically significant.

## Results

### Identification of DEGs

**FIGURE 1:**
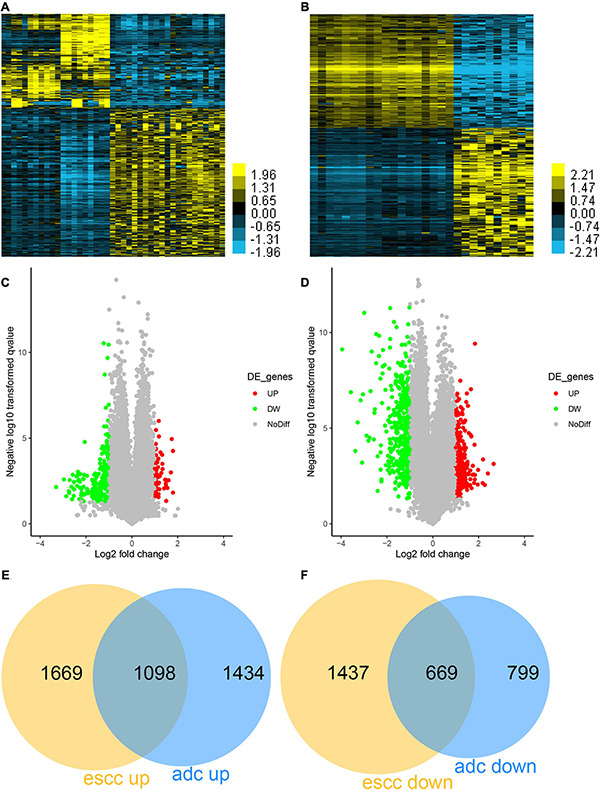
Identification of differentially expressed genes (DEGs) in esophageal adenocarcinoma (EA) and esophageal squamous cell carcinoma (ESCC). **(A)** The differently expressed genes between EA and Barrett’s esophagus samples were shown using Heatmap. **(B)** The differently expressed genes between ESCC and esophageal squamous epithelium samples were shown using Heatmap. **(C)** The differently expressed genes between EA and Barrett’s esophagus samples were shown using Volcano Plot. **(D)** The differently expressed genes between ESCC and esophageal squamous epithelium samples were shown using Volcano Plot. **(E)** The common upregulated genes in both EA and ESCC were determined using Venn diagram. **(F)** The common downregulated genes in both EA and ESCC were determined using Venn diagram.

GSE26886 datasets were used to compare the gene expression among different types of EC. First, 2,667 genes were identified to be induced, and 2,106 genes were identified to be reduced in ESCC compared to esophageal squamous epithelium samples (Figures 1A,C). Meanwhile, 2,532 genes were identified to be induced, and 1,468 genes were identified to be reduced in EA compared to Barrett’s esophagus samples (Figures 1B,D). Finally, we revealed 1,098 common induced genes in both EA and ESCC (Figure 1E) and 669 common reduced genes in both EA and ESCC compared to normal samples (Figure 1F), suggesting that these genes may have a crucial role in the tumorigenesis and progression of both EA and ESCC. Of note, we also found that 1,669 ESCC-specific upregulated, 1,434 EA-specific upregulated, 1,437 ESCC-specific downregulated, and 799 EA-specific downregulated genes, further confirming that that there are significant differences in the pathogenesis between EA and ESCC (Figures 1E,F).

### Bioinformatics Analyses of Common DEGs in EA and ESCC

**FIGURE 2:**
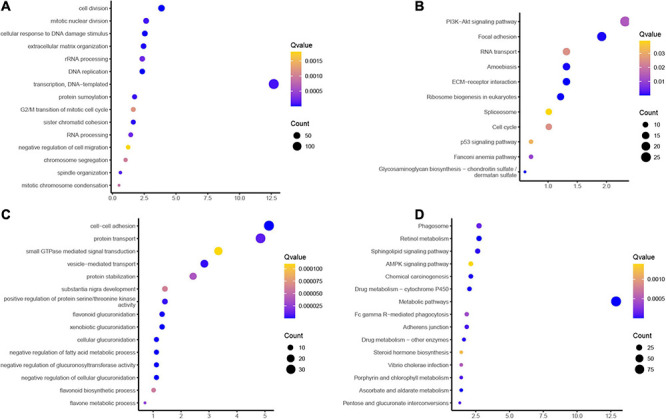
Bioinformatics analyses of common differentially expressed genes (DEGs) in esophageal adenocarcinoma (EA) and esophageal squamous cell carcinoma (ESCC). **(A,B)** Gene Ontology (GO) functions and Kyoto Encyclopedia of Genes and Genomes (KEGG) analysis of the common upregulated genes in esophageal cancer (EC). **(C,D)** GO functions and KEGG analysis of the common downregulated genes in esophageal cancer (EC).

Database for Annotation, Visualization and Integrated Discovery was used for bioinformatics analysis. GO functions analysis results showed that the common induced gene was related to mitotic chromosome condensation, spindle organization, chromosome segregation, negative regulation of cell migration, RNA processing, sister chromatid cohesion, protein SUMOylation, transcription, DNA replication, extracellular matrix organization, cellular response to DNA damage stimulus, and cell division (Figure 2A). The common reduced gene was related to flavone metabolic process, flavonoid biosynthetic process, negative regulation of cellular glucuronidation and fatty acid metabolic process, flavonoid glucuronidation, serine/threonine kinase activity, substantia nigra development, protein stabilization, vesicle-mediated transport, and cell–cell adhesion (Figure 2C).

Kyoto Encyclopedia of Genes and Genomes pathway showed that induced genes were involved in regulating Fanconi anemia pathway, p53 signaling pathway, cell cycle, spliceosome, ribosome biogenesis in eukaryotes, ECM-receptor interaction, amebiasis, RNA transport, focal adhesion, and phosphoinositide 3-kinase (PI3K)–Akt signaling pathway (Figure 2B). Reduced genes were involved in regulating porphyrin and chlorophyll metabolism, *Vibrio cholerae* infection, steroid hormone biosynthesis, adherens junction, Fc gamma R-mediated phagocytosis, metabolic pathways, drug metabolism–cytochrome P450, chemical carcinogenesis, AMP-activated protein kinase (AMPK) signaling pathway, sphingolipid signaling pathway, retinol metabolism, and phagosome (Figure 2D).

### Identification of DEGs Between EA and ESCC

**FIGURE 3:**
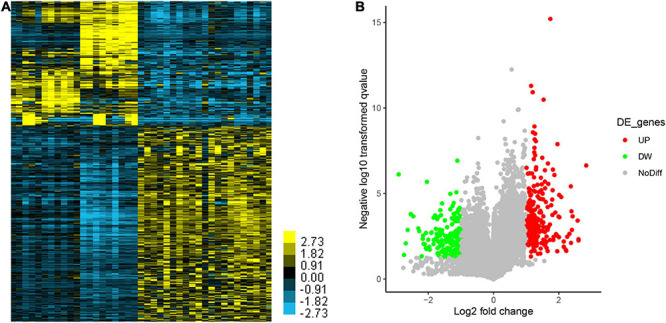
Identification of differentially expressed genes (DEGs) between esophageal adenocarcinoma (EA) and esophageal squamous cell carcinoma (ESCC). **(A,B)** The differently expressed genes between EA and ESCC were shown using **(A)** Heatmap and **(B)** Volcano Plot.

In order to reveal the expression signature that was used to distinguish EA from ESCC, we analyzed the different expression of genes. Finally, we revealed 857 induced genes and 880 reduced genes in EA compared to ESCC samples (Figures 3A,B).

### Bioinformatics Analyses of DEGs Between EA and ESCC Samples

**FIGURE 4:**
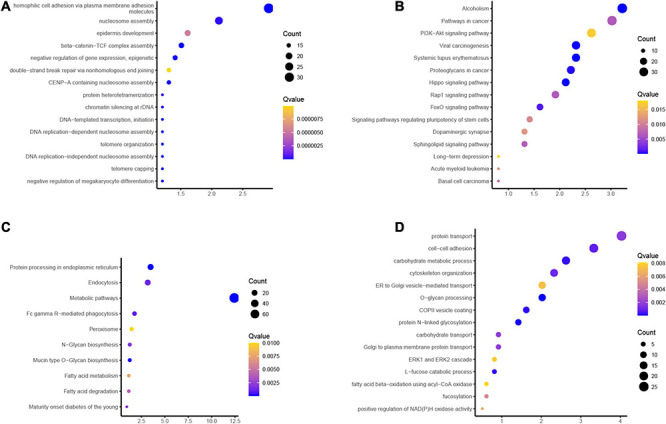
Bioinformatics analyses of differentially expressed genes (DEGs) between esophageal adenocarcinoma (EA) and esophageal squamous cell carcinoma (ESCC) samples. **(A,B)** Gene Ontology (GO) functions and Kyoto Encyclopedia of Genes and Genomes (KEGG) analysis of the upregulated genes in EA compared to ESCC. **(C,D)** GO functions and KEGG analysis of the common downregulated genes in EA compared to ESCC.

GO functions analysis results showed that the induced genes in ESCC were related to telomere capping, nucleosome assembly, telomere organization, DNA-templated transcription, initiation, and chromatin silencing at ribosomal DNA (rDNA) (Figure 4A). KEGG pathway analysis showed induced genes in ESCC were related to the regulation of pluripotency of stem cells, FoxO signaling pathway, Rap1, Hippo, and PI3K–Akt signaling (Figure 4B).

GO functions analysis results showed that the reduced genes in ESCC were related to fatty acid degradation, fatty acid metabolism, Mucin-type O-glycan biosynthesis, N-glycan biosynthesis, peroxisome, Fc gamma R-mediated phagocytosis, metabolic pathways, and endocytosis (Figure 4C). KEGG pathway analysis showed reduced genes in ESCC were related to carbohydrate transport, protein N-linked glycosylation, COPII vesicle coating, O-glycan processing, endoplasmic reticulum (ER) to Golgi vesicle-mediated transport, cytoskeleton organization, carbohydrate metabolic process, and cell–cell adhesion (Figure 4D).

### Identification of Hub Tumor Progression Genes Between EA and ESCC

**FIGURE 5:**
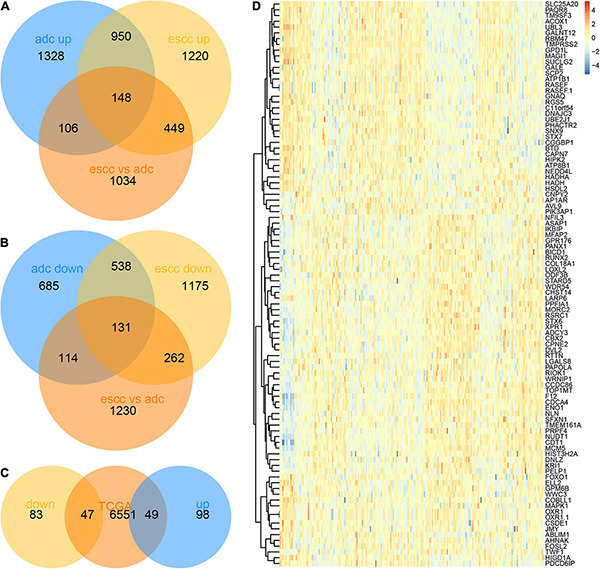
Identification of hub tumor progression genes between esophageal adenocarcinoma (EA) and esophageal squamous cell carcinoma (ESCC). **(A)** One hundred forty-seven common induced genes that was also differently expressed between EA and ESCC were identified using Venn diagram. **(B)** One hundred thirty common reduced genes that was also differently expressed between EA and ESCC were identified using Venn diagram. **(C)** The differently expressed genes between esophageal cancer (EC) and normal samples were shown using heatmap. **(D)** Forty-seven common reduced and 49 common induced genes were also differently expressed in EC samples compared to normal samples using The Cancer Genome Atlas (TCGA) database.

Finally, we identified 148 common induced genes that were also differently expressed between EA and ESCC (Figure 5A) and 131 common reduced genes that were also differently expressed between EA and ESCC (Figure 5B). In order to confirm the expression of these hub genes, we analyzed The Cancer Genome Atlas (TCGA) dataset. As expect, we found that 47 common reduced and 49 common induced genes were also differently expressed in EC samples compared to normal samples using TCGA database (Figures 5C,D).

**FIGURE 6:**
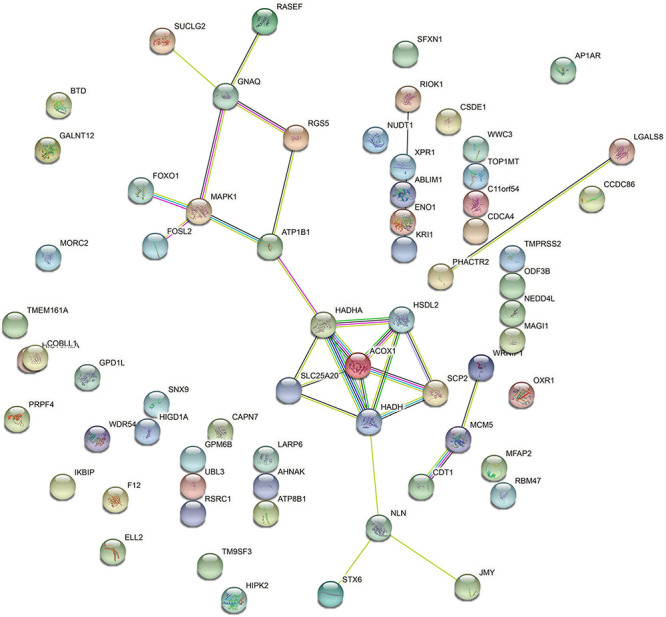
The protein–protein interaction (PPI) network of differentially expressed genes (DEGs) was constructed.

The PPI network of DEGs was further built. Based on PPI network analysis, we identified 10 hub genes with connection > 2, including GNAQ, RGS5, MAPK1, ATP1B1, HADHA, HSDL2, SLC25A20, ACOX1, SCP2, and NLN (Figure 6).

### Validation of Hub Genes and Survival Curve Analysis

**FIGURE 7:**
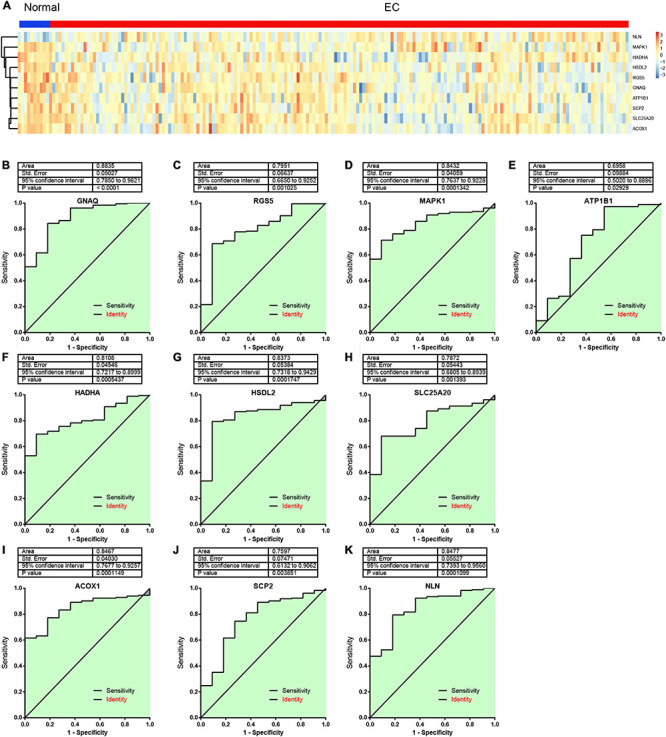
The area under the curve (AUC) analysis of hub genes for distinguishing esophageal cancer (EC) samples from normal tissues. **(A)** The expression levels of hub genes in EC and normal samples were analyzed using The Cancer Genome Atlas (TCGA) database. **(B–K)** The area under the curve (AUC) analysis of GNAQ **(B)**, RGS5 **(C)**, MAPK1 **(D)**, ATP1B1 **(E)**, HADHA **(F)**, HSDL2 **(G)**, SLC25A20 **(H)**, ACOX1 **(I)**, SCP2 **(J)**, and NLN **(K)** for distinguishing EC samples from normal tissues.

Furthermore, we confirmed the expression levels of 10 hub genes using the TCGA dataset. The results showed that GNAQ, SCP2, RGS5, MAPK1, ATP1B1, SLC25A20, HADHA, HSDL2, ACOX1, reduced in EC samples, and NLN were significantly induced in EC samples compared to normal tissues (Figure 7A).

Furthermore, the area under the curve (AUC) of GNAQ for distinguishing EC samples from normal tissues was 0.8835 (Figure 7B). The AUC of RGS5 for distinguishing EC samples from normal tissues was 0.7951 (Figure 7C). The AUC of MAPK1 for distinguishing EC samples from normal tissues was 0.8432 (Figure 7D). The AUC of ATP1B1 for distinguishing EC samples from normal tissues was 0.6958 (Figure 7E). The AUC of HADHA for distinguishing EC samples from normal tissues was 0.8108 (Figure 7F). The AUC of HSDL2 for distinguishing EC samples from normal tissues was 0.8373 (Figure 7G). The AUC of SLC25A20 for distinguishing EC samples from normal tissues was 0.7872 (Figure 7H). The AUC of ACOX1 for distinguishing EC samples from normal tissues was 0.8457 (Figure 7I). The AUC of SCP2 for distinguishing EC samples from normal tissues was 0.7597 (Figure 7J). The AUC of NLN for distinguishing EC samples from normal tissues was 0.8477 (Figure 7K).

**FIGURE 8:**
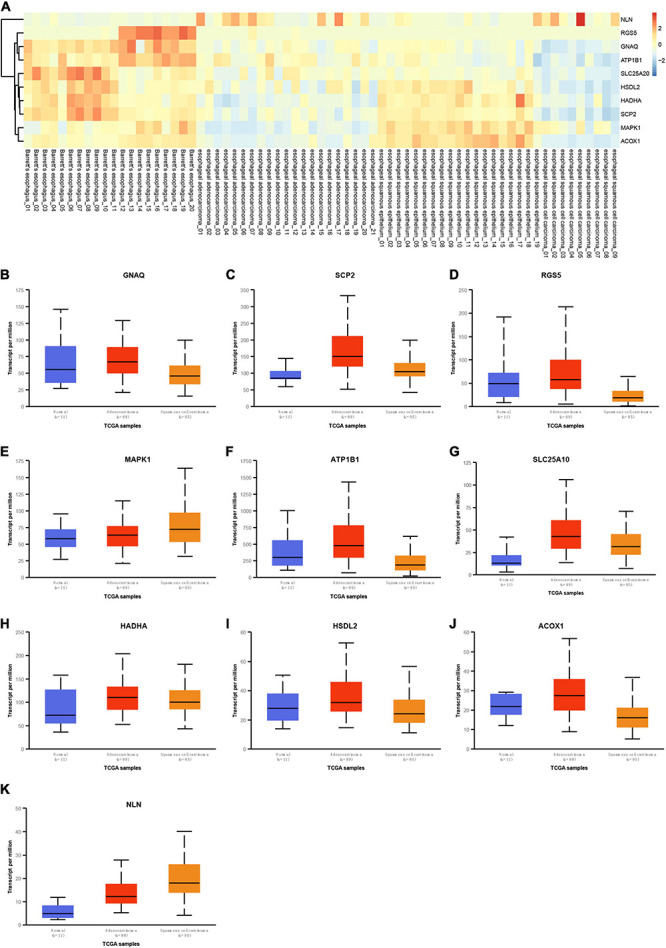
Validation of hub genes expression in esophageal adenocarcinoma (EA) and esophageal squamous cell carcinoma (ESCC). **(A)** GNAQ, RGS5, MAPK1, ATP1B1, HADHA, HSDL2, SLC25A20, ACOX1, and SCP2 were reduced in esophageal cancer (EC) samples, and NLN was significantly induced in EC samples compared to normal tissues by analyzing GSE26886. **(B–K)** GNAQ **(B)**, SCP2 **(C)**, RGS5 **(D)**, MAPK1 **(E)**, ATP1B1 **(F)**, HADHA **(G)**, HSDL2 **(H)**, ACOX1 **(I)**, SLC25A20 **(J)**, and NLN **(K)** were differently expressed in EA and ESCC samples compared to normal tissues by analyzing UALCAN database.

Next, the transcription expression data of hub genes in normal tissues, EA, and ESCC were obtained using UALCAN, which were differently expressed between EA and normal samples and between ESCC and normal samples (Figure 8A). As presented in Figure 8, we found that GNAQ (Figure 8B), SCP2 (Figure 8C), RGS5 (Figure 8D), ATP1B1 (Figure 8F), SLC25A20 (Figure 8G), HADHA (Figure 8H), HSDL2 (Figure 8I), and ACOX1 (Figure 8J) were suppressed in ESCC samples compared to EA samples; however, MAPK1 (Figure 8E) and NLN (Figure 8K) were suppressed in ESCC samples compared to EA samples.

**FIGURE 9:**
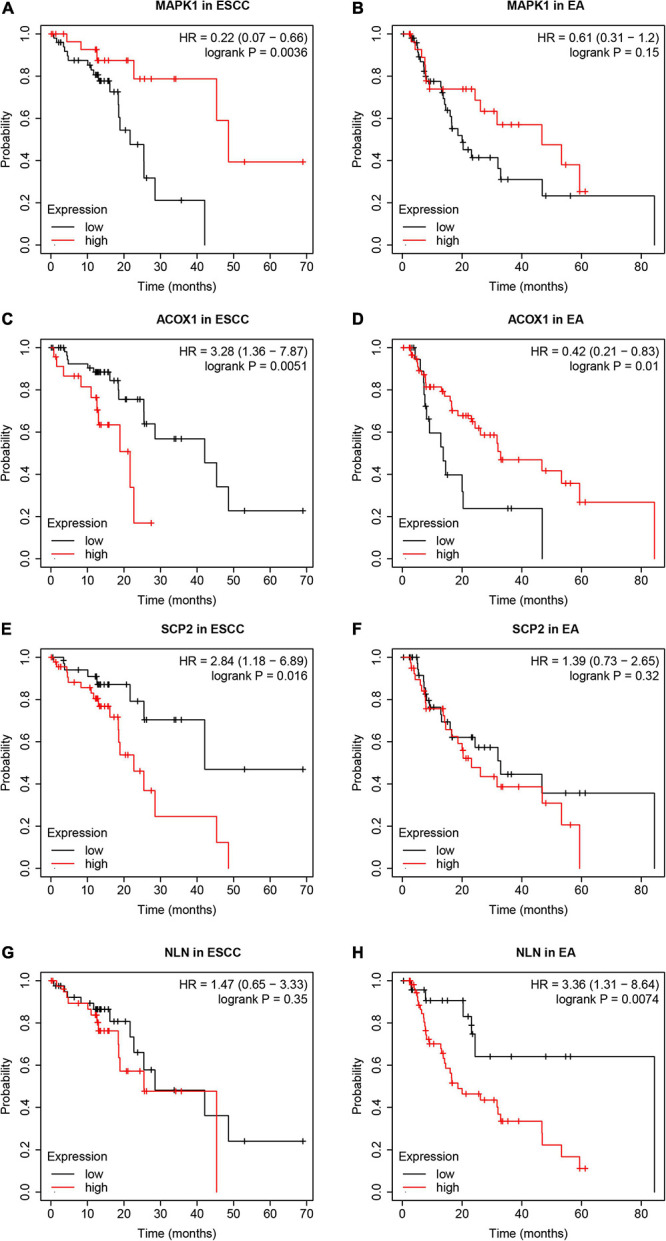
The dysregulation of hub genes was correlated to the survival time in patients with esophageal adenocarcinoma (EA) and esophageal squamous cell carcinoma (ESCC). **(A,B)** Higher expression levels of MAPK1 were associated with longer overall survival (OS) time in patients with ESCC, not EA. **(C,D)** Higher expression levels of ACOX1 were associated with shorter OS time in patients with ESCC and longer OS time in patients with EA. **(E,F)** Higher expression levels of SCP2 were associated with shorter OS time in patients with ESCC, but not EA. **(G,H)** Higher expression levels of NLN were associated with shorter OS time in patients with EA, but not ESCC.

We utilized the Kaplan–Meier Plotter online tool to analyze the correlation between OS time and hub genes expression in EA and ESCC. We found higher expression levels of MAPK1 were related to longer OS time in patients with ESCC, not EA (Figures 9A,B). Higher expression levels of ACOX1 were related to shorter OS time in patients with ESCC and longer OS time in patients with EA (Figures 9C,D). Higher expression levels of SCP2 were related to shorter OS time in patients with ESCC, but not EA (Figures 9E,F). Higher expression levels of NLN were related to shorter OS time in patients with EA, but not ESCC (Figures 9G,H). However, we did not observe a significant correlation between OS time and GNAQ, RGS5, ATP1B1, HADHA, HSDL2, and SLC25A20 (data not shown).

## Discussion

Although there are marked differences in the pathogenesis, the treatment for ESCC and EA are similar, including chemotherapy, radiotherapy, and surgery, in which surgery is the most common treatment (Campbell and Villaflor, 2010). Identifying biomarkers for EC development, progression, and prognosis is essential for understanding EC and improving clinical decision-making. The aim of this study was to identify the similarities and differences between ESCC and EA. In this study, we analyzed GSE26886 datasets and identified 1,098 common induced genes in both EA and ESCC and 669 common reduced genes in both EA and ESCC, indicating that these genes may have a crucial role in EC tumorigenesis and progression. We also revealed 857 induced genes and 880 reduced genes in EA compared to ESCC samples. Furthermore, we conducted bioinformatics analysis to reveal the potential roles of these genes. Finally, we utilized the public databases to verify the levels of hub genes in EC samples. We thought we could provide novel biomarkers for EA and ESCC prognosis.

Over the past decades, multiple efforts were paid to identify the mechanisms involved in regulating EA and squamous cell carcinoma. For example, targeting the thromboxane A2 pathway driven by cox1/2 can inhibit Barrett’s esophagus and EA (Zhang T. et al., 2019). TRIM27 promotes the occurrence and development of esophageal cancer by regulating the PTEN/Akt signaling pathway (Zhang T. et al., 2019). FOXD2-AS1 silencing inhibits the growth and metastasis of esophageal cells by regulating the mir-145-5p/Cdk6 axis (Shi W. et al., 2020). ATP6V0D2 is a subunit related to proton transport, which plays a carcinogenic effect in esophageal cancer and is related to epithelial–mesenchymal transition (Qi et al., 2020). However, there was still a lack of comprehensive analysis of hub signaling in esophagus tumors. In this study, we identified DEGs in esophagus cancer and revealed 1,098 common induced and 669 common reduced genes in both adenocarcinoma and squamous cell carcinoma, which may present the hub mechanisms in esophagus cancers. Bioinformatics analysis found that upregulated genes mainly participated in cell cycle regulation *via* modulating a series bps, including chromosome segregation, sister chromatid cohesion, and DNA replication. The reduced DEGS were involved in regulating metabolism, *via* modulating a series bps, including flavone metabolic process and cellular glucuronidation. Of note, we found several hub signaling, such as p53 and PI3K–Akt signaling pathway. As a multifunctional transcription factor, p53 regulates the expression of more than 2,500 target genes (Stegh, 2012). p53 affects numerous and highly diverse cellular processes, including maintaining genomic stability and fidelity, metabolism, and longevity (Stegh, 2012). It is one of the most important and widely studied tumor suppressors. p53 is activated by various stresses, the most important of which are genotoxic damage, hypoxia, and heat shock (Hsu et al., 1995; Hu et al., 2012). It can block cancer progression by triggering transient or permanent growth arrest, DNA repair, or promoting cell death. This effective and versatile anticancer activity spectrum, together with genomic and mutation analysis, shows that p53 is inactivated in more than 50% of human cancers (Nigro et al., 1989). PI3K signaling pathway is one of the most common signaling pathways in human tumors and plays a key role in the occurrence and development of tumors (Liu et al., 2009).

Esophageal carcinoma includes EA and ESCC. It is one of the most common gastrointestinal cancers, causing about 375,000 deaths worldwide each year. More and more literatures support different treatment strategies according to the histological characteristics of esophageal cancer (Domper Arnal et al., 2015). The different treatment strategies and outcomes of AC and SCC reflect the impact of histology on the natural history and treatment outcomes of some cancers. Therefore, it is an urgent need to identify DEGS between EA and SCC. In this study, we identified 598 induced and 924 reduced genes in squamous cell carcinoma compared to adenocarcinoma samples. Bioinformatics analysis showed that the induced genes in SCC was related to telomere capping, telomere organization, and DNA replication. Telomeres had crucial roles in tumorigenesis by modulating the proliferation and cell cycle of cancer cells (Cacchione et al., 2019). Downregulated genes in SCC was related to fatty acid metabolism and extracellular signal-regulated kinase 1 (ERK1) and ERK2 cascade. ERK signaling is activated in tumors, which was related to regulate multiple processes such as proliferation and survival (Kohno and Pouyssegur, 2006). Previous studies demonstrated that this signaling had a crucial role in both EA and ESCC. For example, Chen et al. (2019) reported that targeting ERK significantly inhibits growth and metastasis of esophageal squamous cell carcinoma cells. Miral R Sadaria et al. (2013) found that suppressing ERK 1/2 activation reduced cell viability and proliferation of human esophageal adenocarcinoma cells. Finally, we identified 147 common induced genes that were also differently expressed between EA and ESCC and 130 common reduced genes that were also differently expressed between EA and ESCC.

Based on PPI network analysis, we identified 10 hub genes with connection > 2, including GNAQ, RGS5, MAPK1, ATP1B1, HADHA, HSDL2, SLC25A20, ACOX1, SCP2, and NLN. Very interestingly, the further confirmation showed that most of these hub genes, including GNAQ, RGS5, MAPK1, ATP1B1, HADHA, HSDL2, SLC25A20, ACOX1, and SCP2, were reduced in EC samples, suggesting that they may play a tumor-suppressive role in EC. Only NLN was report to significantly be overexpressed in EC samples compared to normal tissues. Moreover, we found that GNAQ, RGS5, ATP1B1, HADHA, HSDL2, SLC25A20, ACOX1, and SCP2 were reduced in ESCC samples compared to EA samples; however, MAPK1 and NLN were reduced in ESCC samples compared to EA samples. Among these genes, GNAQ was reported to be related to uveal melanoma progression. GNAQ mutations have led to the activation of several downstream pathways in uveal melanoma, including ERK, p38, c-JUN N-terminal kinase (JNK), and Yap signaling (Shoushtari and Carvajal, 2014). In this study, we found that the expression of GNAQ in esophageal carcinoma and EA was lower than normal. The expression of GNAQ in ESCC was also lower than that in EA. G protein signal transduction regulator 5 (RGS5) is a family of GTPase activators and signal transduction molecules that negatively regulate the function of G protein (Liang et al., 2005). More specifically, RGS5 stops the signal transduction in heterotrimer G protein and is located in plasma membrane and cytoplasm (1). Recently, RGS5 has been identified as a major gene induced in pericytes and is associated with some morphological changes in tumor vasculature. It was found that RGS5 level decreased with the increase in antivascular endothelial growth factor (anti-VEGF) antibody expression as a result of angiogenesis inhibition (Wang et al., 2019).

Of note, this study for the first time revealed that the dysregulation of MAPK1, ACOX1, SCP2, and NLN is significantly correlated to the survival time in EC patients, whose functional importance had been implied in multiple cancer types. MAPK1 belongs to the MAP kinase family (Guo et al., 2020). MAPK1 is a well-known oncogene, which is overexpressed in various types of human cancers, such as lung tumor, ovarian, cervical, and gastric cancer. ACOX1 is an enzyme that catalyzes the first and rate-limiting desaturation of long-chain acyl coenzyme A to 2-*trans*-enol coenzyme A and transfers electrons to the reaction to react with molecular oxygen to form hydrogen peroxide (Zhang et al., 2021). Recent studies have shown that ACOX1 may be involved in tumorigenesis. For example, ACOX1 knockout contributed to liver cancer progression (Chen et al., 2018). In addition, ACOX1 destabilizes p73, thereby inhibiting the intrinsic apoptotic pathway of lymphoma cells and regulating the sensitivity to doxorubicin. SCP2 has no enzyme activity but binds branched chain lipids such as phytic acid and cholesterol derived from phytol (Milligan et al., 2017). SCP2 enhances the uptake and metabolism of branched chain fatty acids (Milligan et al., 2017), which is a recognized intracellular cholesterol transporter, which can direct cholesterol to cholesterol-rich cell membrane microstructure. It has been reported that the expression of SCP2 is related to the progression of glioma, and the suppression of SCP2 protein expression can inhibit the proliferation of tumor cells by inducing autophagy. In addition, SCP2-mediated cholesterol membrane transport promotes pituitary adenoma growth by activating hedgehog signaling (Ding et al., 2019). This study is the first to reveal the important role of SCP2 in esophageal cancer. It may be a potential biomarker for the prognosis of esophageal cancer. NLN is a 78-kDa monomer protein with 704 amino acid residues and only hydrolyzes peptides with 5–17 amino acids (Cavalcanti et al., 2014). *In vivo* studies have shown that NLN is associated with multiple human diseases (Garrido et al., 1999; Massarelli et al., 1999; Rioli et al., 2003). This study is the first to show that NLN is induced in esophageal cancer and has the ability to distinguish between EA and ESCC.

In addition, we should point out several limitations of this study. First, the expression levels of hub genes, such as MAPK1, ACOX1, SCP2, and NLN, were not confirmed using clinical samples. Second, the molecular functions of these hub genes in EC remained largely unclear. Using loss of functions with specific small-interfering RNAs (siRNAs) targeting these hub genes will further strength the findings of this study.

## Conclusion

In this study, we analyzed the GSE26886 dataset and identified 1,098 genes induced in EA and ESCC, and 669 genes were reduced in EC and ESCC, suggesting that these genes may play an important role in the occurrence and development of EC tumors. Bioinformatics analysis showed that these genes were involved in cell cycle regulation, p53 signaling pathway, and PI3K/Akt signaling pathway. In addition, we identified 147 induced genes and 130 reduced genes differentially expressed in EA and ESCC. The expression of ESCC in the EA group was different from that in the control group. By PPI network analysis, we identified 10 hub genes, including GNAQ, RGS5, MAPK1, ATP1B1, HADHA, HSDL2, SLC25A20, ACOX1, SCP2, and NLN. TCGA validation showed that these genes were present in the dysfunctional samples between EC and normal samples and between EA and ESCC. Kaplan–Meier analysis showed that MAPK1, ACOX1, SCP2, and NLN were associated with overall survival in patients with EC. We believe that this study may provide a new biomarker for the prognosis of EA and ESCC.

## Data Availability Statement

The datasets presented in this study can be found in online repositories. The names of the repository/repositories and accession number(s) can be found in the article/supplementary material.

## Author Contributions

SL conceived and designed the study. FW, LZ, and YXu performed the analyses. All authors wrote the manuscript and read and approved the manuscript.

## Conflict of Interest

The authors declare that the research was conducted in the absence of any commercial or financial relationships that could be construed as a potential conflict of interest.

## Footnotes

1 https://string-db.org/

2 http://ualcan.path.uab.edu/
